# Quality of Life in Post-COVID-19 Patients after Hospitalization

**DOI:** 10.3390/healthcare10091666

**Published:** 2022-08-31

**Authors:** Olivera Mitrović-Ajtić, Dejana Stanisavljević, Sanja Miljatović, Teodora Dragojević, Emilija Živković, Miloš Šabanović, Vladan P. Čokić

**Affiliations:** 1 Institute for Medical Research, University of Belgrade, 11000 Belgrade, Serbia; 2 Institute for Medical Statistics and Informatics, Faculty of Medicine, University of Belgrade, 11000 Belgrade, Serbia; 3 Clinic for Infectious and Tropical Diseases, University Clinical Centre of Serbia, 11000 Belgrade, Serbia

**Keywords:** mobility, usual activity, pain, anxiety, EQ-5D, post-COVID-19 patients, quality of life

## Abstract

The COVID-19 pandemic has had a strong impact on people’s quality of life (QoL), which is affected by social and economic changes as well as by mental and physical health. The aim of this study was to determine QoL in post-COVID-19 patients who had required hospitalization, and to identify relevant sociodemographic data. We used questionnaires which considered demographic and socioeconomic data, health and vaccination status, the pandemic situation, and EQ-5D scoring. The interactions of all data and the scores of EQ-5D were analyzed. Multivariate logistic regression analysis was applied to the five dimensions of EQ-5D. In this single-hospital-cohort study, the average times elapsed since initial diagnosis and hospital admission were 2.5 (76.3 ± 18.1 days) and 5 months (155.4 ± 33.9 days), respectively. Post-COVID-19 females were 3–5 times more likely to be affected in terms of anxiety/depression, and in negative impact upon their usual activities, at 5 months after diagnosis. At the same time, reductions in mobility were 3–4 times more likely in elderly post-COVID-19 patients, whose levels of pain and discomfort increased. Single patients, those with low incomes, and those with severe clinical outcomes were 2–4 times more likely to experience a reduction in their usual activities, while the presence of co-morbidities and lower levels of education were associated with increased pain and discomfort. Aging-induced pain/discomfort and anxiety/depression were significantly exacerbated in elderly patients with widespread vaccination. Our study revealed effects of demographic and socioeconomic factors upon lower QoL in post-COVID-19 patients in four dimensions of EQ-5D: mobility, usual activity, pain/discomfort, and anxiety/depression, 5 months after first diagnosis and hospitalization.

## 1. Introduction

Coronavirus disease (COVID-19) is an infectious disease caused by the SARS-CoV-2 virus, as defined by the by World Health Organization. Elderly people and those with co-morbidities such as cardiovascular disease, diabetes, or cancer are more likely to develop serious clinical outcomes after contracting COVID-19. With COVID-19 patients who require hospital admission, associated factors include age, gender, number of prior emergency admissions, being in a palliative phase of life, residency in a nursing home, and multiple morbidities [1]. Post-acute COVID-19 Syndrome (PACS) is a set of symptoms that appear during or after COVID-19 infection, which persist for more than 12 weeks, and cannot be explained by an alternative diagnosis [2]. Many patients have experienced PACS for 3 to 12 months after recovery [3]. One year after acute infection with COVID-19, a fifth of patients exhibited a significant reduction in their quality of life (QoL) [4]. Anxiety and pain or discomfort were the most common problems (33–35%) that affected post-COVID-19 patients’ QoL at the 6- and 12-month follow-up stages [5]. Some meta-analyses have shown the predominant post-COVID-19 symptoms after 12 months to be depression (23%), anxiety (22%), and difficulties with concentration (18%) [6]. In contrast, other researchers have found that those infected with COVID-19 experienced no significant differences in levels of depression and anxiety disorders, compared to an upper-respiratory-infection group [7]. Moreover, regardless of the severity of the initial disease, hospitalized post-COVID-19 patients reported longitudinal improvements in physical and mental health, with most returning to their original work within two years, while health-related quality of life (HRQoL) continued to recover, particularly in terms of anxiety or depression [8]. 

Both hospitalized and non-hospitalized COVID-19 patients with PACS have been found to exhibit anxiety, depression, and sleep disturbance three or more months after the onset of COVID-19 [9]. One study found that older patients reported impaired ability to carry out daily activities (35%), reduced mobility (33%) and more pain or discomfort (33%), six months after hospitalization due to COVID-19 [10]. In addition, post-COVID-19 patients who had been hospitalized reported more problems with mobility, pain or discomfort, and anxiety or depression during a one-year follow-up [11]. Another study found that, almost 4 months after hospital admission, post-COVID-19 patients were impacted in their HRQoL, and in the factors of the European five-dimensional health scale (EQ-5D: mobility, self-care, pain, anxiety or depression, usual activity) [12]. Patients hospitalized with COVID-19 showed lower values of EQ-5D-3L after 3 months, indicating worsening pain/discomfort and anxiety/depression, while females were independently associated with lower HRQoL [13].

Considering the previous observations, the objective of this study was to determine the QoL in post-COVID-19 patients who had been diagnosed and hospitalized 2.5 or 5 months previously using the EQ-5D questionnaire related to demographic and socioeconomic characteristics. We also assessed the impacts of clinical outcome severity, vaccination, and specific co-morbidities in our work. By such means, we interpolated health and social parameters to accurately assess QoL in post-COVID-19 patients.

## 2. Materials and Methods

### 2.1. Data Collection

The study was conducted in the Clinic for Infectious and Tropical Diseases, University Clinical Centre of Serbia, Belgrade, Serbia. The study involved previously hospitalized post-COVID-19 patients, with moderate and severe clinical outcomes (just one at ICU), discharged from the hospital between March 2021 and April 2022. The post-COVID-19 patient survey was conducted amongst patients who had been admitted to hospital either 2.5 or 5 months previously. An informed consent statement was attached to the QoL and EQ-5D questionnaires. Data was collected from patients by means of face-to-face interviews with two healthcare professionals. Additionally, information on sociodemographic and clinical characteristics was extracted from patients’ medical records, including vaccination status, time since diagnosis, and the severity of clinical outcome.

### 2.2. Questionnaires

The first part of the questionnaire was related to demographic and socioeconomic characteristics of the post-COVID-19 patients, including participants’ age categorized into two groups (younger or older than 50 years), sex, marital status, occupation, level of education, income, employment status, level of worry about contracting COVID-19 (5-degree classification), and individual history of chronic illness (Table S1). The post-COVID-19 patients were classified as having no chronic diseases or as having one, two, or more than three such diseases. The second part of the questionnaire assessed the pandemic situation at the time, and the degree to which patients were affected by COVID-19 in terms of their social activity, daily life, sleeping, diet, exercise, working stability, personal learning children’s education, and income; as well as their relationships with parents, friends and spouses. The third part was the HRQoL EQ-5D questionnaire that includes five dimensions (mobility, self-care, usual activities, pain/discomfort, anxiety/depression), each with three levels for assessment of patient health states (no problems, some problems, extreme problems/loss of ability). Quantification of the parameters of the QoL is presented in Table S2.

### 2.3. Data Analysis

Descriptive statistics were obtained from the patient characteristic and outcome measures and expressed as means with standard deviations or as numbers and percentages, as appropriate. Differences between groups were analyzed using Pearson’s chi-squared test and Student’s t-test. In addition, we assessed the relative size of any effect using standardized estimates of effect size according to Cohen’s benchmarks. Univariable and multivariable logistic regressions were used with the five health dimensions as dependent variables (0 = no problems, 1 = some/extreme problems). Results were expressed as odds ratios (OR) and 95% confidence intervals (CI). All tests were two-tailed. *p* < 0.05 was considered statistically significant. The IBM SPSS 25 (Chicago, IL, USA, 2017) package was used for these analyses.

## 3. Results

### 3.1. Demographic Characteristics of Post-COVID-19 Participants

At the outset, our study involved 117 hospitalized post-COVID-19 participants including 77 males and 40 females, with an average age of 59.1 ± 13.6 years. However, just 89 of these completed the QoL survey (Table S1): 84 at 2.5 months after diagnosis (76.3 ± 18.1 days), and 68 at 5 months after diagnosis (155.4 ± 33.9 days). These participants included 63 males (56.2 ± 12.9 years old) and 26 females (61.2 ± 12.6 years old), with no significant differences in age (unpaired t-test, *p* = 0.0984). The demographic characteristics of post-COVID-19 participants are presented in Table 1. Between the two studied samples, there were no significant differences in the proportions of participants who were female (*p* = 0.931), aged over 50 years (*p* = 0.635), married (*p* = 0.900), unemployed (*p* = 0.130), with a chronic disease condition (*p* = 0.838), with university-level education (*p* = 0.309), low-income (*p* = 0.552), with worries about contracting COVID-19 (*p* = 0.942), or with severe clinical outcomes (*p* = 0.334).

Just 42.7% (38) of the participants were vaccinated prior to SARS-CoV2 infection; a majority (76.3 %) received BBIBP-CorV (Sinopharm) vaccine while the rest were vaccinated with Gam-COVID-Vac (Sputnik V, 4), ChAdOx1-S (AstraZeneca COVISHIELD, 2), or BNT162b2 (Pfizer-BioNTech, 3), in line with the BBIBP-CorV vaccination figures for the general public (~62%). A total of 36.8% of participants had received a booster dose. Patients aged over 50 years were 50.8% vaccinated, while patients younger than 50 years were 23.3% vaccinated. Regarding co-morbidities, these were present in a majority (61.8%) of post-COVID-19 patients. The most frequent were arterial hypertension (76.4%) and diabetes mellitus (23.6%). Regarding effects of the pandemic, 26.8% and 28.9% of post-COVID-19 participants were affected after 2.5 and 5 months, respectively, in terms of their social activity, usual activity, sleeping, diet, and exercise (Table 1). These effects were almost equally balanced between negative and positive after 2.5 months, while after 5 months the effect on social activities was predominantly negative (59 ± 15.9 vs. 41 ± 15.9 for positive, *p* = 0.0549). Overall, after 2.5 months, mobility was reduced by 36.9%, self-care by 1.2%, and usual activities by 26.5%, while pain and discomfort rose by 44%, and anxiety and depression rose by 21.4% in the post-COVID-19 patients. After 5 months, mobility was reduced by 35.3%, self-care by 1.5%, and usual activities by 20.6%, while pain and discomfort rose by 40% and anxiety and depression by 28.4% in the post-COVID-19 patients. 

### 3.2. EQ-5D of Post-COVID-19 Participants 2.5 Months after Hospital Admission

We determined the percentage of reported problems for each dimension of EQ-5D in relation to the demographic and socioeconomic characteristics, health status and the pandemic situation of participants 2.5 months after diagnosis and hospitalization. In addition, we observed clinical outcomes and vaccination uptake levels in accordance with EQ-5D (Table 2). Females had four times more anxiety and depression than males (*p* = 0.001), as well as two times more pain/discomfort (*p* = 0.004). Moreover, mobility was three times more likely to be reduced in elderly patients (age over 50 years, *p* = 0.006). Pain and discomfort were three times less common among post-COVID-19 patients with high levels of education (university) compared to those with lower education (*p* = 0.002). A similar outcome was recorded for patients worried about contracting COVID-19 (*p* = 0.007), this time with a two-fold difference compared to their counterparts. Patients with a severe clinical outcome were more than twice as likely to have reduced their usual activities (*p* = 0.019). The calculated effect sizes for differences in all assessed variables ranged from low to large according to Cohen’s guidelines for describing effect sizes (Table S3).

### 3.3. EQ-5D of Post-COVID-19 Participants 5 Months after Hospital Admission

We determined the percentages of reported problems for each dimension of EQ-5D in relation to the demographic and socioeconomic characteristics, health status and the pandemic situation of participants 5 months after diagnosis and hospitalization. In addition, we observed clinical outcomes and vaccination uptake levels with reference to EQ-5D (Table 3). In females, mobility was two times more affected (*p* = 0.031), and usual activities five times more affected than in males (*p* ≤ 0.0001). The incidence of anxiety and depression was also three times higher in females (*p* = 0.001). Reductions in mobility were four times greater in elderly patients (age over 50 years, *p* = 0.017), while single patients were four times more likely to be affected in terms of their usual activities, compared to married patients (*p* = 0.007). Post-COVID-19 patients with co-morbidities were more than twice as likely to experience pain and discomfort as patients without such conditions (*p* = 0.023), while patients with high education (university) were four times less likely to experience pain and discomfort than patients with lower education (*p* = 0.009). Having a low income resulted in a tripling of impact upon usual activities (*p* = 0.047), and severe clinical outcomes also reduced usual activities by a factor of three (*p* = 0.010). Those who were vaccinated were twice as likely to experience pain and discomfort (*p* = 0.011) and two-and-a-half-times more likely to experience anxiety and depression (*p* = 0.029) five months after hospital admission. The calculated effect sizes for differences in all assessed variables ranged from low to large according to Cohen’s guidelines for describing effect sizes (Table S4).

### 3.4. Logistic Regression Models with Health Dimensions as Dependent Variables of Post-COVID-19 Patients 2.5 Months after Hospital Admission

By multivariate logistic regression analysis, we examined relationships between each dimension of EQ-5D and influence factors of the post-COVID-19 patients 2.5 months after hospital admission (Table 4). Age was significantly associated with the mobility dimension (*p* = 0.012), and clinical outcome showed a significant relationship with the usual activities dimension (*p* = 0.022). The multivariable logistic regression demonstrated that clinical outcome (*p* = 0.005), education level (*p* = 0.004), being female (*p* = 0.003), and being worried about contracting COVID-19 (*p* = 0.008) were independent significant predictors of pain and discomfort symptoms (*p* = 0.004). There was also a significant association between being female and the anxiety/depression dimension of QoL (*p* = 0.012).

### 3.5. Logistic Regression Models with Health Dimensions as Dependent Variables of Post-COVID-19 Patients 5 Months after Hospital Admission

By multivariate logistic regression analysis, we examined relationships between each dimension of EQ-5D and influence factors of the post-COVID-19 patients 5 months after diagnosis and hospital admission (Table 5). Age was significantly associated with the mobility dimension (*p* = 0.036), while severe clinical outcome, female sex (*p* = 0.003) and low income (*p* = 0.015) showed significant relationships with the usual activities dimension. In the multivariable regression model, age (*p* = 0.036) and education level (*p* = 0.033) were significantly associated with pain. Being female was the only significant predictor of anxiety and depression (*p* = 0.002) in post-COVID-19 patients, 5 months after diagnosis and admission.

## 4. Discussion

Previous studies reported that patients with COVID-19 in the early period after discharge had reduced QoL, higher incidences of anxiety, and more severe anxiety symptoms, particularly women [14]. In our study, females had an incidence of anxiety and depression three-to-four times higher than that of males 5 months after diagnosis and admission. Female sex and advanced age were the factors most related to lower HRQoL in post-COVID-19 patients who required hospitalization, while other factors included the presence and number of comorbidities, high body mass index, lower levels of education, and unemployment [15]. We found that pain and discomfort were more pronounced in women 2.5 months after hospital admission, while after 5 months, mobility was twice as likely to be affected in women, and usual activities five times more likely. In line with these findings, and with previous reports, we might suggest that women are at risk of reduced HRQoL in the post-COVID-19 period. However, men are more likely to experience severe manifestations of COVID-19 and are at higher risk of death from the disease [16]. Furthermore, epidemiological studies have shown that males experience higher case fatality rates after infection with SARS-CoV-1 and Middle East respiratory syndrome coronavirus (MERS-CoV) [17,18]. Another study found that residual CT abnormalities were common in hospitalized COVID-19 patients one year after recovery, especially fibrotic changes in severe and critical cases [19]. COVID-19 affects multiple organs and systems such as the respiratory, cardiovascular, gastrointestinal, endocrine, hematological, urogenital, and nervous systems. This multisystemic effect may make women more susceptible to the long-term effects of COVID-19. This suggestion is in line with lower female immune response to milder forms of COVID-19, in comparison with males who experience more severe clinical outcomes directly related to a strong immune response.

The authors of [20] showed that female sex, advanced age and co-morbidities were the most reported factors connected with low levels of QoL, regardless of the time elapsed since discharge [20]. We demonstrated that reduced mobility three-to-four times more likely in elderly post-COVID-19 patients 5 months after diagnosis. Moreover, the impact upon usual activities of elderly patients was greater after 2.5 months, as was the impact upon pain levels increased after 5 months. Post-COVID-19 patients with co-morbidities had twice the likelihood of pain and discomfort at 5 months. In addition, after 5 months, those with a severe clinical outcome were two-to-three times more likely to have reduced their usual activities. We can say, therefore, that advanced age, co-morbidities and severe clinical outcomes are responsible for deterioration in HRQoL in the post-COVID-19 period. Further expanded studies of hospitalized post-COVID-19 patients should also involve other COVID-19-related clinical and laboratory parameters as potential markers of disease progression and to assess impact on QoL.

The influenza (H1N1)2009 pandemic had a temporary but significant impact on the HRQoL (EQ-5D) of the majority of patients who required hospital admission, after 5 months [21]. Both influenza outpatients and inpatients reported problems on all five dimensions measured in the EQ-5D, such as pain/discomfort (71.8%), anxiety/depression (62–75.1%), mobility, self-care, and usual activity (below 40%) [22]. The authors of [23] found that HRQoL levels followed the clinical course of Guillain-Barré Syndrome in cases of Zika virus infection, with poorer scores in the early months, followed by recovery within the first year, and stabilization after one year. Another study showed that not having vaccinations for COVID-19 and influenza is associated with poor QoL [24]. Therefore, QoL levels recovered over time after viral infections, but reduced QoL caused by COVID-19 in terms of anxiety or depression still persisted after two years [8]. Regarding reductions in HRQoL for both sexes after influenza (H1N1)2009, statistically significant differences (0.48 female vs. 0.36 male, p<0.05) were recorded only among unemployed outpatients according to the EQ-5D index [21]. In contrast, being female was significantly associated with reduced EQ-5D scores for HRQoL in a study of influenza A outpatients and inpatients [22]. We cannot generally account for the susceptibility of females to reduced HRQoL in all post-infection states, but a certain tendency nevertheless exists.

COVID-19 patients had a lower physical QoL one month after discharge [25]. The influence of COVID-19 on physical HRQoL may resolve over time, but not when it affects daily activities, work, and social activities [26]. The pandemic produced negative impacts on sleep quality, HRQoL and depression symptoms [27]. In our study, we showed that those individuals worried about contracting COVID-19 were twice as likely to experience pain and discomfort 2.5 months after diagnosis. Regarding pandemic effects, the post-COVID-19 patients experienced largely negative effects in terms of their social activities after 5 months. This more than two-fold negative difference lasted up to 5 months for social activities, daily living, sleep, and exercise; and prevailed over time for work stability, personal learning, and income.

Married people, especially women, those with low levels of education, and those currently out of work or with a below-average financial condition reported lower QoL during the pandemic [28]. Low-income and single patients were three-to-four times more likely to report a reduction in usual activities five months after diagnosis, in line with the findings of our study. In addition, a high level of education was associated with a reduced incidence of pain and discomfort in post-COVID-19 patients after five months. Socioeconomic factors greatly influenced the QoL of post COVID-19 patients, and contributed consistently to their recovery. 

Previous studies reported that COVID-19 vaccine improved the physical QoL in participants aged below 50 years, but not their mental QoL, while neither mental nor physical QoL were significantly linked with vaccine status for those aged over 50 years [24]. In our study, vaccinated individuals were twice as likely to experience pain/discomfort and anxiety/depression, 5 months after COVID-19 diagnosis. However, it should be noted that aging is very close to being a statistically significant factor by itself, in terms of increased pain/discomfort (*p* = 0.072) and anxiety/depression (*p* = 0.080) after 5 months (Table 3), and this is confirmed for pain/discomfort (*p* = 0.036) by multivariate analysis (Table 5). In our cohort of post-COVID-19 patients, the majority of vaccinated individuals were aged over 50 years (81.1%). Therefore, we might consider increased pain/discomfort and anxiety/depression as the results of aging, rather than as side-effects of a beneficial vaccination. 

Our study has some limitations, such as a small number of participants and a short timeframe of patient follow-up after COVID-19. It will be interesting to extend the follow-up of this cohort, up to one year, to further assess persistence of symptoms or sequelae. Moreover, in addition to COVID-19 patients with moderate and severe clinical outcomes, it may be worthwhile to consider ICU patients during any extended follow-up study.

## 5. Conclusions

Understanding the QoL of post-COVID-19 patients may clarify prognoses and provide important indicators of the benefits and risks associated with disease management. In addition, QoL data can identify stages of disease progression that might be introduced into clinical practice to assess outcome severity in therapeutic research. Our study revealed reduced QoL of hospitalized post-COVID-19 patients in four dimensions of EQ-5D: mobility, usual activity, pain/discomfort, and anxiety/depression. The reduced QoL was influenced by demographic and socioeconomic factors such as sex, age, co-morbidities (usually hypertension and diabetes mellitus), education level, income, and the severity of clinical outcome. This evaluation validates the importance of demographic and socioeconomic factors, in addition to medical issues, as risk factors in post-COVID-19 recovery. 

## Figures and Tables

**Table 1:** Characteristics of quality of life of examined COVID-19 patients 2.5 months and 5 months after hospitalization.

Quality of Life after	2.5 Months		5 Months	
	N	%	N	%
**Sex**				
Male	60	69.8	47	69.1
Female	26	30.2	21	30.9
**Age (year)**				
≤50	29	34.5	21	30.8
>50	55	65.5	47	69.2
**Marital status**				
Married	61	72.6	50	73.5
Unmarried	10	11.9	10	14.7
Divorced/widowed	13	15.5	8	11.8
**Employment status**				
Employed	62	72.9	37	54.4
Retired	10	11.8	26	38.2
Unemployed	13	15.3	5	7.4
**Chronic disease condition**				
None	33	38.4	25	36.8
With 1	26	30.2	22	32.3
With 2	10	11.6	7	10.3
With ≥ 3	17	19.8	14	20.6
**Education level, school**				
Primary	3	3.6	1	1.5
Junior	44	52.4	37	54.4
Senior	11	13.1	14	20.6
University	26	31	16	23.5
**Family income**				
Low	3	3.5	1	1.5
Lower	10	11.8	7	10.4
Middle	61	71.8	52	77.6
Higher	10	11.8	7	10.4
High	1	1.2		
**Worry about contracting COVID-19**				
Very high	9	10.6	7	10.3
High	34	40	27	39.7
Low	39	45.9	29	42.6
Very low	3	3.5	5	7.4
**Pandemic effects**				
Yes	26.8 ± 11	31.6 ± 12.8	19.2 ± 9.3	28.9 ± 13.8
No	57.7 ± 10.3	68.4 ± 12.8	46.8 ± 8.5	71.1 ± 13.8
**Clinical outcome**				
Moderate	57	67.9	51	75
Severe	27	32.1	17	25

**Table 2:** Percentage of post-COVID-19 patients in 5 dimensions of EQ-5D 2.5 months after hospital admission.

EQ-5D	Mobility			Self-Care			Usual Activities			Pain/Discomfort			Anxiety/Depression		
	None	Some	*p* Value	None	Some	*p* Value	None	Some	*p* Value	None	Some	*p* Value	None	Some	*p* Value
**Sex**															
Male	67.8	32.2	0.174	98.3	1.7	0.518	77.6	22.4	0.203	66.1	33.9	**0.004**	88.1	11.9	**0.001**
Female	52	48		100	0		64	36		32	68		55	44	
**Age (year)**															
≤50	84.6	15.4	**0.006**	96.2	3.8	0.136	88	12	0.50	65.4	34.6	0.249	84.6	15.4	0.372
>50	53.4	46.6		100	0		67.2	32.8		51.7	48.3		75.9	24.1	
**Marital status**															
Single	68.2	31.8	0.570	100	0	0.555	68.2	31.8	0.516	54.5	45.5	0.879	77.3	22.7	0.865
Married	61.3	38.7		98.4	1.6		75.4	24.6		56.5	43.5		79	21	
**Employment status**															
Employed, retired	64.1	35.9	0.855	98.7	13	0.802	75.3	24.7	0.086	56.4	43.6	0.877	78.2	21.8	0.926
unemployed	60	40		100	0		40	60		60	40		80	20	
**Chronic disease condition**															
No	75.8	24.2	0.068	100	0	0.420	78.8	21.2	0.352	63.6	36.4	0.301	81.8	18.2	0.535
At least one	56	44		98	2		69.4	30.6		52	48		76	24	
**Education level**															
University	76.9	23.1	0.080	100	0	0.506	84.6	15.4	0.124	80.8	19.2	**0.002**	75.9	24.1	0.372
other	56.9	45.8		98.3	1.7		68.4	31.6		44.8	55.2		84.6	15.4	
**Income level**															
other	63.4	36.6	0.902	98.6	1.4	0.671	75.7	24.3	0.293	59.2	40.8	0.171	78.9	21.1	0.877
low	61.5	38.5		100	0		61.5	38.5		38.5	61.5		76.9	23.1	
**Worry about contracting COVID-19**															
No	73.2	26.8	0.063	97.6	2.4	0.309	82.5	17.5	0.075	70.7	29.3	**0.007**	82.9	17.1	0.348
Yes	53.5	46.5		100	0		65.1	34.9		41.9	58.1		74.4	25.6	
**Pandemic effects**															
No	62.5	37.5	0.944	100	0	0.530	87.5	12.5	0.067	62.5	37.5	0.451	91.7	8.3	0.066
Yes	63.3	36.7		98.3	1.7		67.8	32.2		53.3	46.7		73.3	26.7	
**Clinical outcome**															
Moderate	62.9	37.1	0.952	98.4	1.6	0.555	80.3	19.7	**0.019**	50	50	0.066	74.2	25.8	0.103
Severe	63.6	36.4		100	0		54.5	45.5		72.7	27.3		90.9	9.1	
**Vaccine**															
No	66	34	0.509	98	2	0.413	77.6	22.4	0.321	62	38	0.180	82	18	0.359
Yes	58.8	41.2		100	0		67.6	32.4		47.1	52.9		73.5	26.5	

Bolded *p* values represent statistical significance.

**Table 3:** Percentage of post-COVID-19 patients in 5 dimensions of EQ-5D 5 months after hospital admission.

EQ-5D	Mobility			Self-Care			Usual Activities			Pain/Discomfort			Anxiety/Depression		
	None	Some	*p* Value	None	Some	*p* Value	None	Some	*p* Value	None	Some	*p* Value	None	Some	*p* Value
**Sex**															
Male	74.5	25.5	**0.031**	97.9	2.1	0.508	89.4	10.6	**≤0.001**	65.2	34.8	0.187	84.8	15.2	**0.001**
Female	47.6	52.4		100	0		52.4	47.6		47.4	52.6		47.6	52.4	
**Age (year)**															
≤50	88.9	11.1	**0.017**	100	0	0.552	88.9	11.1	0.197	77.8	22.2	0.072	88.9	11.1	0.080
>50	58	42		98	2		74	26		53.2	46.8		67.3	32.7	
**Marital status**															
Single	55.6	44.3	0.273	100	0	0.552	55.5	44.4	**0.007**	52.9	47.1	0.497	61.1	38.9	0.184
Married	70	30		97.4	2.6		86	11.4		62.5	37.5		77.6	22.4	
**Employment status**															
Employed, retired	67.2	32.8	0.488	8.4	1.6	0.805	78.1	21.9	0.886	59.7	40.3	0.813	73	27	0.932
unemployed	50	50		100	0		75	25		66.7	33.3		75	25	
**Chronic disease condition**															
No	76.9	23.1	0.145	96.2	3.8	0.206	80.8	19.2	0.664	76.9	23.1	**0.023**	80.8	19.2	0.268
At least one	59.5	40.5		100	0		76.2	23.8		48.7	51.3		68.3	31.7	
**Education level**															
University	81.3	18.8	0.149	100	0	0.583	87.5	12.5	0.299	87.5	12.5	**0.009**	87.5	12.5	0.142
other	61.5	38.5		98.1	1.9		77	25		51	49		68.6	31.4	
**Income level**															
other	69.5	30.5	0.076	98.3	1.7	0.716	81.4	18.6	**0.047**	63.2	36.8	0.306	75.9	24.1	0.127
low	37.5	62.5		100	0		50	50		42.9	57.1		50	50	
**Worry about contracting COVID-19**															
No	67.6	32.4	0.801	97.1	2.9	0.321	82.4	17.6	0.388	66.7	33.3	0.272	76.5	23.5	0.539
Yes	64.7	35.3		100	0		73.5	26.5		56	44		69.7	30.3	
**Pandemic effects**															
No	64.7	35.3	0.965	100	0	0.560	82.4	17.6	0.569	41.2	58.8	0.088	82.4	17.6	0.289
Yes	65.3	34.7		98	2		75.5	24.5		65.2	34.8		68.8	31.3	
**Clinical outcome**															
Moderate	69.6	30.4	0.197	98.2	1.8	0.647	83.9	16.1	**0.010**	58.2	41.8	0.491	74.5	25.5	0.584
Severe	50	50		100	0		50	50		79	30		66.7	33.3	
**Vaccine**															
No	65.8	34.2	0.941	100	0	0.264	78.9	21.1	0.825	74.3	25.7	**0.011**	83.8	16.2	**0.029**
Yes	66.7	33.3		96.7	3.3		76.7	23.3		43.3	56.7		60	40	

Bolded *p* values represent statistical significance.

**Table 4:** Multivariate logistic regression analysis on the relationships between dimensions of EQ-5D and influence factors in post-COVID-19 patients 2.5 months after hospital admission.

Dimensions of EQ-5D	Influence Factors	Beta		*p* Value	Odds Ratio	95%CI
Mobility	Age	0.713		0.012	4.613	1.409–15.103
Usual activities	Clinical outcome	0.544		0.022	3.403	1.191–9.725
Pain/discomfort	Female sex	0.875		0.003	6.702	1.894–23.723
	Clinical outcome	0.900		0.005	0.131	0.032–0.541
	Education level	0.935		0.004	0.134	0.034–0.524
	Worry	0.758		0.008	4.511	1.483–13.725
Anxiety/depression	Female sex	0.811		0.002	5.837	1.911–17.824

**Table 5:** Multivariate logistic regression analysis on the relationships between dimensions of EQ-5D and influence factors in post-COVID-19 patients 5 months after hospital admission.

Dimensions of EQ-5D	Influence Factors	Beta		*p*	Odds Ratio	95%CI
Mobility	Age	0.742		0.036	5.431	1.120–26.333
Usual activities	Female sex	1.083		0.003	10.139	2.174–47.286
	Income	0.820		0.015	12.298	1.627–92.952
	Clinical outcome	0.699		0.034	6.108	1.143–32.647
Pain/discomfort	Age	0.698		0.036	1.056	1.004–1.111
	Education level	-0.785		0.033	0.167	0.032–0.865
Anxiety/depression	Female sex	0.847		0.002	6.129	1.893–19.845

## Supplementary Materials

The following supporting information can be downloaded at: https://www.mdpi.com/article/10.3390/healthcare10091666/s1, Table S1: Questionnaire for the Quality of Life of the post COVID-19 patients; Table S2: Quantification of the parameters of the Quality of life of the post COVID-19 patients; Table S3: Effect sizes with 95% confidence interval for 5 dimensions of EQ-5D in post-COVID-19 patients after 2.5 months of hospital admission; Table S4: Effect sizes with 95% confidence interval for 5 dimensions of EQ-5D in post-COVID-19 patients after 5 months of hospital admission.
