# Supplementary material for: Quality of Life in Post-COVID-19 Patients after Hospitalization
Source: Healthcare (Basel). 2022 Aug 31;10(9):1666. doi: 10.3390/healthcare10091666 (PMC9498792; doi:10.3390/healthcare10091666)
Supplement: Supplementary file 1 [file healthcare-10-01666-s001.zip › healthcare-1884132-supplementary.pdf]

Table S1. Questionnaire for the Quality of Life of the post-COVID-19 patients.
The questionnaires included demographic and socioeconomic data, health status, the condition epidemic situation and EQ-5D scale.

1. Your sex: ☐male ☐female

2. Your age: _____(year)

3. Your marital status: ☐married ☐unmarried ☐divorced ☐widowed

4. Your occupation: ☐In service ☐retirement ☐in home ☐no job

5. Your education level: ☐primary school education and below ☐high school

☐higher school ☐university and above

6. What is the level of your household income locally?

☐highest ☐high ☐normal ☐low ☐lowest

7. Are you worried that you will contract this disease?

☐very worried ☐worried ☐not worried ☐not worried at all

8. Have you suffered from a chronic disease in the past 6 months: ☐Yes ☐No

If you have a chronic disease, which of the following types (multiple choices) :
☐hypertension ☐diabetes ☐dyslipidemia ☐cerebrovascular disease
☐heart disease (coronary heart disease, arrhythmia, heart valve disease) ☐bronchiectasis
☐peripheral vascular disease ☐chronic obstructive pulmonary ☐asthma
☐pulmonary fibrosis ☐viral hepatitis ☐fatty liver ☐other chronic liver diseases
☐gallbladder disease ☐gastritis ☐gastric ulcer ☐duodenal ulcer ☐chronic enteritis ☐intestinal polyps ☐chronic pain ☐gout ☐arthritis ☐thyroid disease ☐kidney disease ☐bladder disease ☐ uterine and ovarian disease ☐prostate disease ☐otitis media
☐deafness☐glaucoma ☐cataract ☐other eye diseases ☐skin diseases
☐blood diseases ☐parkinson's disease ☐other diseases

9. The influence of the "COVID-19" on you in the following aspects:

contents	great negative	negative	a little negative	no	a little positive	positive	great positive
social activities							
daily life and schedule							
sleep							
diet							
exercise							

working stability							
personal learning or creation							
income							
relationship with parents							
relationship with friends							
marriage relationship							
children education							

European five-dimensional health scale (EQ-5D)

Please indicate in the following set of options which statement best reflects your health status today, and type√ in the space.

Mobility

I can mobile around without any difficulty. ☐
I am a little inconvenient. ☐
I'm sick in bed. ☐

Self-Care

I can take care of myself without any difficulty. ☐
I have some difficulties in washing my face, brushing my teeth, bathing or dressing. ☐
I can’t wash my face, brush my teeth, take a bath or dress myself. ☐

Usual activities (such as work, study, housework, family or leisure activities)

I can do usual activities without any difficulty. ☐
I have some difficulties in usual activities. ☐
I can't do usual activities. ☐

Pain/Discomfort

I don't have any pain or discomfort. ☐
I feel moderate pain or discomfort. ☐
I feel extreme pain or discomfort. ☐

Anxiety/Depression

I don't feel anxious or depressed. ☐
I feel moderate anxiety or depression. ☐
I feel extremely anxious or depressed. ☐

Table S2. Quantification of the parameters of the Quality of life of the post-COVID-19 patients.

**Demographic and socioeconomic data, and health status**										
marital status	married	1	unmarried	2	divorced	3	widowed	4		
occupation	in service	1	retirement	2	in home	3	no job	4		
education /school	primary	1	high	2	higher	3	university	4		
income	highest	1	high	2	normal	3	low	4	minimal	5
disease contract worry	very worried	1	worried	2	not worried	3	not worried at all	4		
chronic disease (n)	Quantity from the list (each disease is 1 point)									

	great negative	negative	a little negative	no	a little positive	positive	great positive
social activities	1	2	3	4	5	6	7
daily life, schedule	1	2	3	4	5	6	7
sleep	1	2	3	4	5	6	7
diet	1	2	3	4	5	6	7
exercise	1	2	3	4	5	6	7
working stability	1	2	3	4	5	6	7
personal learning or creation	1	2	3	4	5	6	7
income	1	2	3	4	5	6	7
Relationship with parents	1	2	3	4	5	6	7
relationship with friends	1	2	3	4	5	6	7
marriage relationship	1	2	3	4	5	6	7
children education	1	2	3	4	5	6	7

**European five-dimensional health scale (EQ-5D)**						
Mobility	without any difficulty	0	a little inconvenient	1	in bed	2
Self-Care	without any difficulty	0	some difficulties	1	dependent	2
Usual activities	without any difficulty	0	some difficulties	1	no	2
Pain/Discomfort	no	0	moderate	1	extreme	2
Anxiety/Depression	no	0	moderate	1	extreme	2

Table S3. Effect sizes with 95% confidence interval for 5 dimensions of EQ-5D in post-COVID-19 patients after 2.5 months of hospital admission.

**EQ-5D**	**Mobility**<br>**t ; df**<br>**MD (95%CI)**<br>**d**	**Self-care**<br>**t ; df**<br>**MD (95%CI)**<br>**d**	**Usual activities**<br>**t ; df**<br>**MD (95%CI)**<br>**d**	**Pain / discomfort**<br>**t ; df**<br>**MD (95%CI)**<br>**d**	**Anxiety / depression**<br>**t ; df**<br>**MD (95%CI)**<br>**d**
**Sex, female**	t=1.371;df=82<br>0.158<br>(-0.071-0.387)<br>d=0.33	t=0.649;df=82<br>-0.017<br>(-0.069-0.035)<br>d=0	t=1.284;df=81<br>0.136<br>(-0.075-0.346)<br>d=0.31	t=2.996;df=82<br>0.341<br>(0.115-0.567)<br>d=0.71	t=3.473;df=82<br>0.321<br>(0.137-0.505)<br>d=0.75
**Age >50**	t=2.833;df=82<br>0.312<br>(0.093-0.531)<br>d=0.73	t=1.505;df=82<br>-0.038<br>(-0.089-0.012)<br>d=0	t=1.989;df=81<br>0.208<br>(0.000-0.415)<br>d=0.51	t=1.161;df=82<br>0.137<br>(-0.097-0.371)<br>d=0.26	t=0.897;df=82<br>0.088<br>(-0.107-0.282)<br>d=0.22
**Marital status**	t=0.570;df=82<br>0.069<br>(-0.172-0.310)<br>d=0.14	t=0.593;df=82<br>0.016<br>(-0.038-0.070)<br>d=0	t=0.652;df=81<br>-0.072<br>(-0.293-0.148)<br>d=0.15	t=0.153;df=82<br>-0.019<br>(-0.267-0.229)<br>d=0.02	t=0.171;df=82<br>-0.018<br>(-0.223-0.187)<br>d=0.05
**Employment status**	t=0.183;df=81<br>0.041<br>(-0.405-0.487)<br>d=0.08	t=0.252;df=81<br>-0.013<br>(-0.114-0.089)<br>d=0	t=1.738;df=80<br>0.353<br>(-0.051-0.758)<br>d=0.71	t=0.155;df=81<br>-0.036<br>(-0.496-0.425)<br>d=0.08	t=0.093;df=81<br>-0.018<br>(-0.401-0.365)<br>d=0.05
**Chronic disease condition**	t=1.849;df=81<br>0.198<br>(-0.015-0.410)<br>d=0.43	t=0.811;df=81<br>0.020<br>(-0.029-0.069)<br>d=0	t=0.936;df=80<br>0.094<br>(-0.106-0.294)<br>d=0.23	t=1.041;df=81<br>0.116<br>(-0.106-0.339)<br>d=0.24	t=0.623;df=81<br>0.058<br>(-0.128-0.244)<br>d=0.15
**Education level**	t=1.770;df=82<br>-0.200<br>(-0.425-0.025)<br>d=0.43	t=0.667;df=82<br>-0.017<br>(-0.069-0.034)<br>d=0	t=1.554;df=81<br>-0.162<br>(-0.369-0.045)<br>d=0.40	t=3.216;df=82<br>-0.359<br>(-0.582to-0.137)<br>d=0.79	t=0.897;df=82<br>-0.088<br>(-0.282-0.107)<br>d=0.22
**Income level**	t=0.125;df=82<br>0.018<br>(-0.275-0.311)<br>d=0.02	t=0.426;df=82<br>-0.014<br>(-0.080-0.052)<br>d=0	t=1.058;df=81<br>0.142<br>(-0.125-0.408)<br>d=0.30	t=1.381;df=82<br>0.207<br>(-0.091-0.505)<br>d=0.42	t=0.156;df=82<br>0.020<br>(-0.230-0.269)<br>d=0.05
**Worry about get COVID-19**	t=1.886;df=82<br>0.197<br>(-0.011-0.404)<br>d=0.40	t=1.024;df=82<br>-0.024<br>(-0.072-0.023)<br>d=0	t=1.807;df=81<br>0.174<br>(-0.018-0.365)<br>d=0.39	t=2.751;df=82<br>0.289<br>(0.080-0.497)<br>d=0.60	t=0.944;df=82<br>0.085<br>(-0.094-0.264)<br>d=0.22
**Epidemic effects**	t=0.071;df=82<br>-0.008<br>(-0.243-0.226)<br>d=0.02	t=0.630;df=82<br>0.017<br>(-0.036-0.069)<br>d=0	t=1.860;df=81<br>0.197<br>(-0.014-0.408)<br>d=0.46	t=0.758;df=82<br>0.092<br>(-0.149-0.332)<br>d=0.18	t=1.866;df=82<br>0.183<br>(-0.012-0.379)<br>d=0.51
**Clinical outcome**	t=0.060;df=82<br>-0.007<br>(-0.248-0.234)<br>d=0.03	t=0.593;df=82<br>-0.016<br>(-0.070-0.038)<br>d=0	t=2.402;df=81<br>0.258<br>(0.044-0.471)<br>d=0.54	t=1.861;df=82<br>-0.227<br>(-0.470-0.016)<br>d=0.48	t=1.649;df=82<br>-0.167<br>(-0.369-0.035)<br>d=0.45
**Vaccine**	t=0.663;df=82<br>0.072<br>(-0.144-0.287)<br>d=0.14	t=0.823;df=82<br>-0.020<br>(-0.068-0.028)<br>d=0	t=0.999;df=81<br>0.099<br>(-0.098-0.296)<br>d=0.22	t=1.353;df=82<br>0.149<br>(-0.070-0.369)<br>d=0.30	t=0.922;df=82<br>0.085<br>(-0.098-0.267)<br>d=0.18

t: t-value; df: degree of freedom; MD: mean difference; d: effect size.

Table S4. Effect sizes with 95% confidence interval for 5 dimensions of EQ-5D in post-COVID-19 patients after 5 months of hospital admission.

EQ-5D	Mobility<br>t ; df<br>MD (95%CI)<br>d	Self-care<br>t ; df<br>MD (95%CI)<br>d	Usual activities<br>t ; df<br>MD (95%CI)<br>d	Pain / discomfort<br>t ; df<br>MD (95%CI)<br>d	Anxiety / depression<br>t ; df<br>MD (95%CI)<br>d
**Sex, female**	t=2.207;df=66<br>0.268<br>(0.026-0.511)<br>d=0.54	t=0.666;df=66<br>-0.021<br>(-0.085-0.043)<br>d=0	t=3.674;df=66<br>0.370<br>(0.169-0.571)<br>d=0.87	t=1.334;df=63<br>0.178<br>(-0.089-0.446)<br>d=0.36	t=3.404;df=65<br>0.372<br>(0.154-0.590)<br>d=0.83
**Age >50**	t=2.444;df=66<br>0.309<br>(0.057-0.561)<br>d=0.74	t=0.597;df=66<br>0.020<br>(-0.047-0.087)<br>d=0	t=1.303;df=66<br>0.149<br>(-0.079-0.377)<br>d=0.39	t=1.829;df=63<br>0.246<br>(-0.023-0.514)<br>d=0.34	t=1.779;df=65<br>0.215<br>(-0.026-0.457)<br>d=0.54
**Marital status**	t=1.104;df=66<br>-0.144<br>(-0.406-0.117)<br>d=0.29	t=0.597;df=66<br>0.020<br>(-0.047-0.087)<br>d=0	t=2.782;df=66<br>-0.304<br>(-0.523to-0.086)<br>d=0.68	t=0.683;df=63<br>-0.096<br>(-0.375-0.184)<br>d=0.18	t=1.344;df=65<br>-0.164<br>(-0.409-0.080)<br>d=0.37
**Employment status**	t=0.697;df=66<br>0.172<br>(-0.320-0.664)<br>d=0.32	t=0.248;df=66<br>-0.016<br>(-0.141-0.110)<br>d=0	t=0.144;df=66<br>0.031<br>(-0.402-0.464)<br>d=0.07	t=0.238;df=63<br>-0.070<br>(-0.657-0.518)<br>d=0.13	t=0.086;df=65<br>-0.020<br>(-0.483-0.444)<br>d=0.04
**Chronic disease condition**	t=1.476;df=66<br>0.174<br>(-0.061-0.409)<br>d=0.27	t=1.277;df=66<br>-0.038<br>(-0.099-0.022)<br>d=0	t=0.437;df=66<br>0.046<br>(-0.164-0.255)<br>d=0.23	t=2.333;df=63<br>0.282<br>(0.041-0.524)<br>d=0.24	t=1.116;df=65<br>0.125<br>(-0.098-0.348)<br>d=0.15
**Education level**	t=1.459;df=66<br>-0.197<br>(-0.467-0.073)<br>d=0.42	t=0.552;df=66<br>-0.019<br>(-0.089-0.050)<br>d=0	t=1.047;df=66<br>-0.125<br>(-0.363-0.113)<br>d=0.31	t=2.688;df=63<br>-0.365<br>(-0.636to-0.094)<br>d=0.83	t=1.488;df=65<br>-0.189<br>(-0.442-0.065)<br>d=0.44
**Income level**	t=1.805;df=65<br>0.320<br>(-0.034-0.674)<br>d=0.65	t=0.366;df=65<br>-0.017<br>(-0.109-0.076)<br>d=0	t=2.028;df=65<br>0.314<br>(0.005-0.622)<br>d=0.66	t=1.031;df=62<br>0.203<br>(-0.190-0.596)<br>d=0.39	t=1.544;df=64<br>0.259<br>(-0.076-0.593)<br>d=0.53
**Worry about get COVID-19**	t=0.253;df=66<br>0.029<br>(-0.203-0.262)<br>d=0.06	t=1.00;df=66<br>-0.029<br>(-0.088-0.029)<br>d=0	t=0.869;df=66<br>0.088<br>(-0.114-0.291)<br>d=0.19	t=1.107;df=63<br>0.135<br>(-0.109-0.380)<br>d=0.28	t=0.618;df=65<br>0.068<br>(-0.151-0.287)<br>d=0.13
**Epidemic effects**	t=0.044;df=64<br>-0.006<br>(-0.278-0.266)<br>d=0.0	t=0.586;df=64<br>0.020<br>(-0.049-0.090)<br>d=0	t=573;df=64<br>0.068<br>(-0.170-0.307)<br>d=0.14	t=1.734;df=61<br>-0.240<br>(-0.518-0.037)<br>d=0.49	t=1.070;df=63<br>0.136<br>(-0.118-0.390)<br>d=0.30
**Clinical outcome**	t=1.302;df=66<br>0.196<br>(-0.105-0.498)<br>d=0.40	t=0.460;df=66<br>-0.018<br>(-0.095-0.060)<br>d=0	t=2.667;df=66<br>0.339<br>(0.085-0.593)<br>d=0.75	t=o.693;df=63<br>-0.118<br>(-0.459-0.222)<br>d=0.48	t=0.551;df=65<br>0.079<br>(-0.207-0.364)<br>d=0.21
**Vaccine**	t=0.075;df=66<br>-0.009<br>(-0.243-0.225)<br>d=0.02	t=1.128;df=66<br>0.033<br>(-0.026-0.092)<br>d=0	t=0.222;df=66<br>0.023<br>(-0.182-0.228)<br>d=0.05	t=2.634;df=63<br>0.310<br>(0.075-0.544)<br>d=0.65	t=2.232;df=65<br>0.238<br>(0.025-0.451)<br>d=0.54

t: t-value; df: degree of freedom; MD: mean difference; d: effect size.
